# Editorial: Recent advances in understanding Tourette syndrome, tic disorders and functional tics

**DOI:** 10.3389/fpsyt.2023.1185489

**Published:** 2023-03-30

**Authors:** Amanda K. Ludlow, Seonaid Anderson, Tammy Hedderly, Kevin J. Black, Christine A. Conelea

**Affiliations:** ^1^Department of Psychology and Sports Sciences, University of Hertfordshire, Hatfield, United Kingdom; ^2^Neuro-Diverse.org, Brussels, Belgium; ^3^Tic and Neurodevelopmental Movements Service (TANDeM), Guy's King's and Saint Thomas' School of Medicine, London, United Kingdom; ^4^Departments of Psychiatry, Neurology, Radiology and Neuroscience, Washington University in St. Louis, St. Louis, MO, United States; ^5^Department of Psychiatry and Behavioral Sciences, Masonic Institute for the Developing Brain, University of Minnesota, Minneapolis, MN, United States

**Keywords:** Tourette syndrome, tic disorders, functional tics, interventions, diagnosis

The goal of this Research Topic was to present recent advances in our knowledge surrounding tic disorders. Tic disorders (TDs) are complex neurological conditions characterized by involuntary, persistent vocalizations and motor movements called tics. While tics are commonly associated with a diagnosis of Tourette syndrome, tics can appear as symptoms of other diagnoses, including an Unspecified Tic Disorder amongst others (1). Tics and functional tic-like behaviors (FTLBS) both appear repetitive and without appropriate context, and they can co-exist. However, compared to Tourette-related tics, FTLBS are often associated with a sudden onset and more complex movements. FTLBS are also associated with higher rates of anxiety and self-harm along with higher female predominance and a later age of onset (2).

Since the beginning of the COVID-19 pandemic, clinicians have noted a marked increase in presentations of these sudden and new onset of FTLBS (3). However, many misconceptions exist surrounding their source. Martindale and Mink provided an overview of how a series of pre-disposing factors, the psychological burden of the COVID-19 pandemic, as well as the rise in the use of social media and digital technology, may be implicated in the rise and spread of FTLBs. The wide-ranging impact of these FLTBS on daily life is also noted, with increases in school absenteeism along with disengagement with education. Using ten case studies, Owen et al. have highlighted that even in the absence of formal therapy, if young people with FTLBs are well-supported with the use of certain techniques, they can manage well at school.

There is strong evidence demonstrating that individuals with tic disorders experience a lower quality of life, with tics shown to have a pervasive impact on all aspects of daily living (4). For example, in Bamigbade et al. mothers revealed tics to be a barrier to positive mealtime experiences, affecting the child's ability to sit, drink and eat. Tics were also found to affect the geniality of mealtimes, with families often avoiding eating out of the home environment due to the challenging and stressful experience of navigating the tics in public. Importantly, Taylor et al. noted the nature of the tics themselves to also have an impact on individuals with TS quality of life, with tics reported to be both physically and psychologically painful. The authors stress the need to understand tic-related pain in the long-term management of tic disorders.

Those with a tic disorder may have heightened awareness of their pain thresholds due to the interoceptive sensibility. For example, Narapareddy et al. reported on altered Interoceptive Sensibility in Adults with Chronic Tic Disorder (CTD), with increased anxiety-associated somatization and increased general body awareness shown. Importantly, in adults with CTD, anxiety-associated somatization was found to be more closely associated with females and obsessive-compulsive symptoms.

Obsessive-compulsive disorder and tic disorders can often co-occur, with individuals frequently presenting with distinct symptoms of CTD and/or OCD (5). However, there are also a subset of individuals with a condition which has been referred to as Tourettic OCD (TOCD), where patients show a specific overlap in tics, compulsions, and their preceding premonitory urges. Katz et al. reviewed the mounting evidence and suggested TOCD has its own distinct phenomenology including an earlier age of onset, male predominance, and specific symptom clusters.

Regarding treatment and management of tics, the European Society for the study of Tourette Syndrome (ESSTS) and the American Academy of Neurology have written guidelines for the management of TS recommending behavior therapy (BT) as a first-line intervention when psychoeducation alone is insufficient (6). Two approaches, habit reversal training (HRT; and its expanded version, Comprehensive Behavioral Intervention for Tics; CBIT) and exposure with response prevention (ERP), have gathered the strongest empirical support. However, a lack of trained therapists, pressures on already overstretched healthcare systems, treatment cost, and travel distance can impact on the availability of face-to-face treatment, with a lack of accessibility being more marked in non-English-speaking countries (7). Reflecting on barriers to treatment, Inoue et al. addressed the preliminary efficacy, feasibility, and acceptability of remotely administered group CBIT (RG-CBIT) in Japan. Positive findings were reported from all three children diagnosed with TS, with all showing a reduction in the severity of tics. Prato et al., also found online remote therapy to be as effective as face-to-face delivery in treatment of the severity of tics, levels of anxiety and obsessive-compulsive symptoms.

Khan et al. provided a synthesis of the research outlining the use of digitally delivered, remote therapy for tics, with promising evidence shown for reduction in the severity of tics in children, young people and adults. With the collective research on digital interventions so far demonstrating good adherence and engagement, although further research is required to understand its cost-effectiveness. However, it is also important to note that less than 40% of adults with TS respond well to CBIT. Ramsey et al. suggest urge intolerance to be one factor that might interact with treatment success. Given the predictive relationship premonitory urges has between tic severity and tic impairment, the authors argue that targeting urge intolerance to improve treatment response is an avenue for future research.

The role of gut bacteria in the symptomology of TS, as well as the possible use of prebiotics in the management of symptoms has recently been discussed. Wang et al. found the abnormal composition of gut microbiota to differentiate children with tic disorders from those without. High levels of Prevotella and Odoribacteis were identified in the tic disorder group, both of which have been associated with symptoms of irritable bowel syndrome (IBS-D), and pediatric autoimmune neuropsychiatric disorders associated with streptococcal infections (PANDAS) respectively. Importantly healthy gut bacteria are essential to allow the body to absorb nutrients as well as influencing food cravings (8). Given Smith and Ludlow findings of high levels of food responsiveness and emotional overeating reported in the TS group, the role of gut bacteria in diet may warrant further exploration.

The work presented in this Research Topic emphasizes the complexity of tic disorders and their impact on everyday life. The future of digital interventions may offer an exciting new avenue to increase accessibility to treatment for those with a tic disorder.

## Author contributions

All authors listed have made a substantial, direct, and intellectual contribution to the work and approved it for publication.
